# Chitosan-Based In Situ Gels for Ocular Delivery of Therapeutics: A State-of-the-Art Review

**DOI:** 10.3390/md16100373

**Published:** 2018-10-09

**Authors:** Teodora Irimia, Cristina-Elena Dinu-Pîrvu, Mihaela Violeta Ghica, Dumitru Lupuleasa, Daniela-Lucia Muntean, Denisa Ioana Udeanu, Lăcrămioara Popa

**Affiliations:** 1 Department of Physical and Colloidal Chemistry, Faculty of Pharmacy, University of Medicine and Pharmacy “Carol Davila”, Bucharest 020956, Romania; teodora.irimia@drd.umfcd.ro (T.I.); mihaela.ghica@umfcd.ro (M.V.G.); lacramioara.popa@umfcd.ro (L.P.); 2 Department of Pharmaceutical Technology and Biopharmacy, Faculty of Pharmacy, University of Medicine and Pharmacy ”Carol Davila”, Bucharest 020956, Romania; dlupuleasa@yahoo.com; 3 Department of Analytical Chemistry and Analysis of Medicines, Faculty of Pharmacy, University of Medicine and Pharmacy of Târgu Mureş, Târgu Mureş 540138, Romania; daniela.muntean@umftgm.ro; 4 Department of Clinical Laboratory and Food Safety, Faculty of Pharmacy, University of Medicine and Pharmacy “Carol Davila”, Bucharest 020956, Romania; denisaudeanu@gmail.com

**Keywords:** chitosan, mucoadhesion, in situ gels, ocular delivery, sol–gel transition

## Abstract

Ocular in situ gels are a promising alternative to overcome drawbacks of conventional eye drops because they associate the advantages of solutions such as accuracy and reproducibility of dosing, or ease of administration with prolonged contact time of ointments. Chitosan is a natural polymer suitable for use in ophthalmic formulations due to its biocompatibility, biodegradability, mucoadhesive character, antibacterial and antifungal properties, permeation enhancement and corneal wound healing effects. The combination of chitosan, pH-sensitive polymer, with other stimuli-responsive polymers leads to increased mechanical strength of formulations and an improved therapeutic effect due to prolonged ocular contact time. This review describes in situ gelling systems resulting from the association of chitosan with various stimuli-responsive polymers with emphasis on the mechanism of gel formation and application in ophthalmology. It also comprises the main techniques for evaluation of chitosan in situ gels, along with requirements of safety and ocular tolerability.

## 1. Introduction

Topical instillation is the most commonly used ocular drug delivery method, but the bioavailability of the active substances is less than 5% [1]. This is due to anatomical and physiological barriers such as nasolacrimal drainage, blinking reflex, corneal factors, protein and melanin binding, or enzymatic metabolism of the drugs [2]. Increased ocular contact time strategies have led to drawbacks such as blurred vision in case of ointments [3] or lack of patient compliance for inserts [4]. A promising alternative are in situ gelling systems that undergo a phase transition in the eye, resulting a gel in response to environmental changes. These systems associate in the same formulation the advantages of solutions such as accuracy and reproducibility of dosing, or ease of administration with the increased retention time of an ointment [5,6].

**Figure 1:**
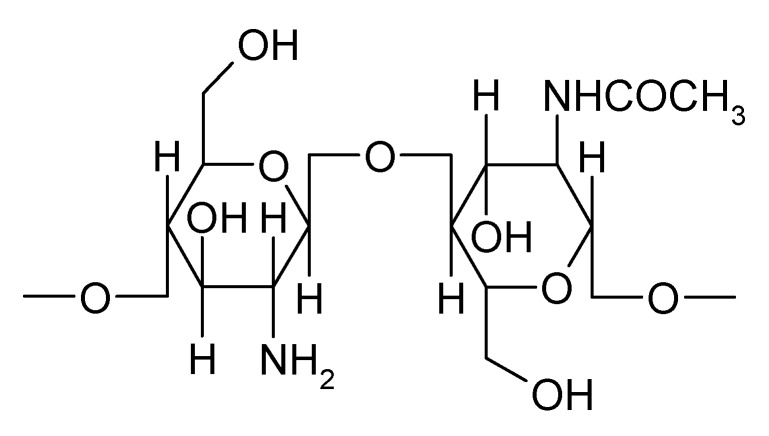
Chemical structure of chitosan.

Natural polymers are widely used not only in the food industry but also in pharmaceutical technology because they are less toxic, biocompatible, and biodegradable. Incorporation of a therapeutic agent into a natural polymeric matrix protects the active compound from degradation, enhances absorption, improves the therapeutic effect, and decreases the frequency of administration. Chitosan is a polymer used as an excipient for delivering different therapeutic agents with applications in drug targeting, nanotechnology, delivery vaccines, or gene therapy. Chitosan, a natural polymer obtained by deacetylation of chitin, consists of repeating units of N-acetyl-D-glucosamine and D-glucosamine linked by β-1,4-glycosidic bonds (Figure 1) [7].

Chitosan is a suitable candidate in ophthalmic formulations due to its biocompatibility, biodegradability, mucoadhesive character, permeation enhancement and corneal wound healing effects, and antimicrobial and antifungal properties. It has pseudoplastic and viscoelastic characteristics that do not disturb the tear film [8,9].

In order to satisfy the features of a gel, chitosan network must fulfill two conditions: the chain interactions to be strong enough to form semi-permanent junction points in the macromolecular network and this should favor the access and residence of water molecules in the polymer network [10].

Chitosan may turn into gel due to physical, chemical, or coordination linkages. Formation of physical gels is the result of non-covalent, reversible interactions such as electrostatic, hydrophobic, or hydrogen bonds. This polymer is capable of forming gel alone, without additives [11]. Acidic solutions of chitosan exposed to alkaline medium determine the lowering of apparent polymer density and appearance of physical gels due to hydrophobic interactions and hydrogen bonds [12].

Chitosan forms in situ gels with negatively charged macromolecules: proteins (gelatin, collagen), polysaccharides (hyaluronic acid, alginate, xanthan, dextran sulfate) or synthetic polyanions (polyacrylic acid). Thermosensitive gels can be obtained by addition of polyol salts such as disodium salt of glycerol phosphate [11]. Because of the polycationic nature, it forms gels with anionic polyelectrolytes through ionic interactions. The solubility of the resulting complexes depends on the net charge. If the net charge is 0, the complex will be insoluble and will precipitate. Although they are biocompatible, physical gels have low mechanical strength, and can react to environmental changes such as temperature, pH, or ionic strength.

Chemical gels are the result of covalent bonds and have resistance to environmental factors. Among the cross-linking agents used, there is glutaraldehyde or genipin. Cross-linking of chitosan is necessary to improve properties such as stability and durability for drug delivery [13]. Chemically cross-linked gels have excellent mechanical properties and good control over the network size of pores, with the disadvantage of using toxic cross-linking agents that require removal. Physical gels offer the advantage of avoiding toxic reagents and are a suitable method for short-term delivery due to low mechanical strength and uncontrolled in vivo dissolution [14].

In situ gelling systems are used to deliver bioactive compounds by instillation into the eye, which upon exposure to the ocular media, change to gel [15].

These formulation-containing polymers are liquid at room temperature (25 °C) and shift to gel after administration because of changes in temperature, pH, or ionic strength [16]. Depending on the physiological mechanisms that produce the gelation of polymers, there are three major categories of polymers: pH triggered in situ gelling polymers, temperature triggered in situ gelling polymers, and ion triggered in situ gelling polymers [17].

## 2. Chitosan-Based In Situ Gels as Carriers for Prolonged Ophthalmic Drug Delivery

### 2.1. pH-Responsive Ocular In Situ Gels Based on Chitosan

**Figure 2:**
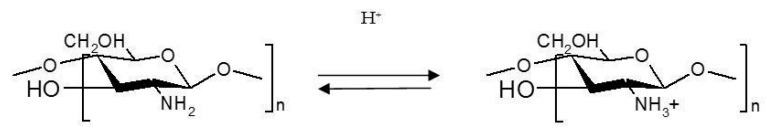
Protonation of chitosan in acidic medium.

pH-responsive gels change their physical and chemical properties at certain pH values due to acidic or basic groups in the polymer structure. Acidic groups are deprotonated in alkaline medium, while basic groups are protonated at acidic pH [18]. Because of its cationic nature, chitosan exhibits a sol–gel transition at pH 6.5 when the medium changes from slightly acidic to neutral. When pH increases, chitosan is deionized and it generates a three-dimensional network [19]. Thus, chitosan gels swell to acidic pH because amino groups are protonated with the appearance of repulsions between polymeric chains (Figure 2) Anionic gels such as those based on carboxymethyl chitosan swell in basic medium due to ionization of acidic groups [20].

The cationic amino group from the chitosan structure leads to the appearance of electrostatic interactions with anionic groups of other polyanions. This determines the formation of polyelectrolyte complexes with non-permanent structures. These hydrogels are well tolerated, biocompatible, and susceptible to environmental change [21]. Hydrogels involving polyelectrolytes are sensitive to pH variations which make functional groups ionized with the appearance of gelling [22]. Thus, chitosan may interact with water-soluble macromolecules such as anionic polysaccharides, dextran sulfate, collagen, or anionic polymers such as polyacrylic acid [10].

**Figure 3:**
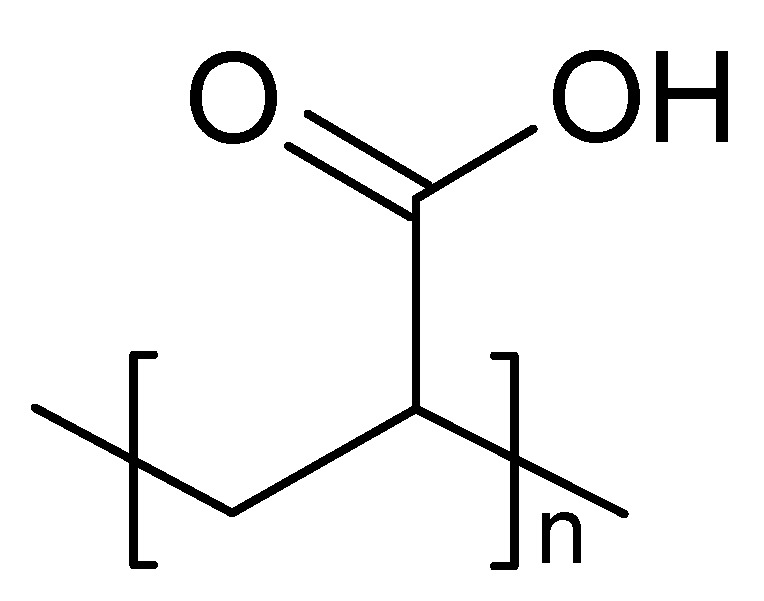
Chemical structure of Carbopol (Polyacrilic acid).

Carbopol is a polyacrylic acid derivative (Figure 3) having a sol–gel transition in aqueous solution when the pH of the medium increases above 5.5. It is non-toxic and non-irritating to humans following topical application. However, the concentration required to form a gel results in acidic solutions that cannot be rapidly neutralized by lacrimal fluid buffers [23]. As a pH-sensitive polymer, the increase of pH results in electrostatic repulsions between anionic groups with the appearance of gelation [24].

The main mechanism of forming complexes between chitosan and Carbopol consists of electrostatic interactions between the amino groups of chitosan and the carboxyl groups of Carbopol [21].

When the chitosan solution is mixed with a Carbopol solution, a highly strength gel is formed under physiological conditions. Gupta and Vyas developed an ophthalmic in situ gel based on gelling ability of the chitosan-Carbopol mixture in simulated lacrimal fluid at pH 7.4 and studied the influence of the preparation on experimentally induced intraocular pressure. According to the authors, this gel increased the ocular retention time and released timolol maleate over 24 h [25]. Unlike conventional drops that are applied twice a day, the advantage is a decrease in frequency of administration of timolol at once daily, improving patients’ compliance [26].

Mucoadhesive polymer, Carbopol enhances the mechanical strength of the formulations and the contact time with the eye surface [27]. The mechanism underlying the mucoadhesive capacity consists in the interaction of polyacrylic acid and mucin with the appearance of electrostatic and hydrophobic interactions, hydrogen bonds, and inter-diffusion [28].

The mucoadhesive properties of Carbopol are associated with those of chitosan, facilitating contact time with the ocular surface [29]. Zaki et al. formulated an ophthalmic in situ gel based on chitosan and Carbopol 940 in which they incorporated ketorolac tromethamine. It prolonged ocular contact time, improving the healing rate of ocular ulcers in rabbits compared to conventional eye drops [30].

Dextran is a branched polymer produced by various strains of bacteria from sucrose, and consists of D-glucose units with α-1,6-linkages and branches at α-1,3-bonds. It is biocompatible and biodegradable [31]. Having similar chitosan properties but negative charge, dextran forms electrolytic complexes with chitosan without requiring difficult synthesis steps or toxic organic solvents [32]. Chavan et al. proposed a formulation of chitosan and dextran sulfate in order to deliver ciprofloxacin in simulated tear fluid at pH 7.4. The association of chitosan-dextran sulfate promoted the efficiency of entrapped ciprofloxacin to approximately 83% and its release in 21 h [33].

### 2.2. Thermoresponsive Ocular In Situ Gels Based on Chitosan

An in situ thermosensitive gel is in the form of a liquid at low temperature and shifts to gel under the influence of temperature [34]. The temperature at which the sol–gel transition occurs is called lower critical solution temperature (LCST). At a temperature below LCST, the hydrogen bonds between the hydrophilic groups of the polymer and the water molecules facilitate dissolution of the polymer chains, and the system remains in the solution. As the temperature rises above the LCST, hydrogen bonding breaks up with the occurrence of hydrophobic interactions in favor of sol–gel transition [35]. An ophthalmic in situ gel should have a phase transition temperature higher than room temperature and become gel at precorneal temperature (35 °C). This avoids refrigerant conditioning and cold administration of the preparation that may be irritating to the eye [34].

**Figure 4:**
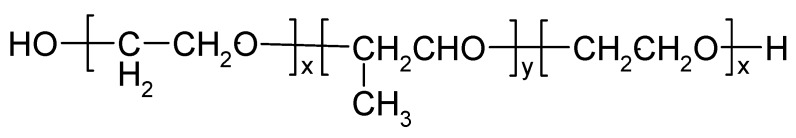
Chemical structure of poloxamer (Pluronic^®^).

Poloxamers, known as Pluronics^®^, are triblock copolymers made up of hydrophilic polyoxyethylene units and hydrophobic polyoxypropylene units (Figure 4).

It exhibits amphiphilic character and thermosensitive behavior [36]. At concentrations of 18% (w/w) or more in aqueous solution, poloxamer 407 is converted from a low viscosity solution to a non-crosslinked hydrogel at ambient temperature [37]. Thermogelation is the result of hydrophobic interactions between polymer chains. When the temperature rises, the poloxamer chains form a micellar structure due to the dehydration of the hydrophobic polyoxypropylene units [38]. Poloxamer gels have several drawbacks: low retention time, poor mechanical properties and high permeability, so they only apply to short-term implantable systems or as excipients for the solubilization of hydrophobic drugs [39].

Gupta et al. formulated an in situ gel based on chitosan and poloxamer for the ocular release of timolol maleate, an antiglaucomatous drug. Thermosensitive polymer, Pluronic F-127, acted as a gelling agent, and chitosan, a pH-sensitive polymer, acted as a permeability enhancer. Chitosan increased the transcorneal permeability of timolol. It had a bioadhesive character, a viscous nature, and a gel-forming ability at ocular pH 7.4. In vitro transcorneal permeability studies were performed on goat cornea and showed an increased permeation after four hours for the in situ gelling system compared to eye drops. That was due to the fact that chitosan had the ability to increase the transmucosal permeability [40].

A study proposed by Varshosaz et al. showed that Pluronic 15% lost its gelling capacity after lacrimal fluid dilution when used alone. Phase change temperature (PCT) turned significantly from 39 °C to 43 °C and its concentration was no longer sufficient for gelling. Pluronic F127 gels were the consequence of hydrogen bonds between the poloxamer ether oxygen atom and water protons. The addition of chitosan which had amino groups increased the number of hydrogen bonds and the mechanical strength of the gel. The formulation containing 15% Pluronic and 0.1% chitosan as a viscosity enhancer was liquid at non-physiological conditions (pH 4, 25 °C) and shifted to gel under physiological conditions (pH 7.4, 37 °C). It could be considered a viable alternative to eye drops for ocular delivery of ciprofloxacin [41].

A prolonged delivery system useful in the treatment of ocular disorders was proposed by Gratieri et al., who considered a combination of poloxamer and chitosan in various proportions, with enhanced mechanical and mucoadhesive properties. According to the results, the solution containing 16% poloxamer had a sol–gel transition temperature around 32 °C. Chitosan used in concentrations ranging from 0.5 to 1.5% (w/w) did not significantly alter the gelation temperature in all concentration ranges analyzed and also contributed to the elasticity of the formulations. The result was possibly due to an effect on diffusion coefficients in the gel structure, favoring the accommodation of unbound water which resulted from dehydration of the micelle core. It has therefore facilitated cross-linking and has helped to increase system elasticity [27].

**Figure 5:**
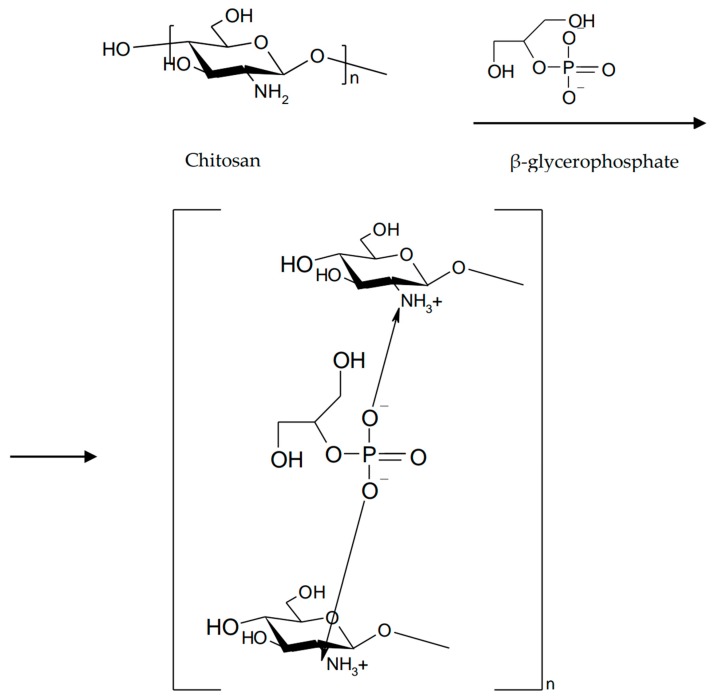
Neutralization of chitosan with β-glycerophosphate.

Gels prepared by neutralization of chitosan with salts of polyols exhibit thermoreversible gelling properties [10]. Chitosan is soluble at pH 6.2 due to protonation of amino groups. The addition of a base will increase the pH but will reduce the electrostatic interactions between the polymer chains that lead to a gel structure. Neutralization of a chitosan solution with a weak base such as β-glycerophosphate (β-GP) maintains the system in solution at physiological pH and at room temperature, but will turn into gel by heating to physiological temperature (37 °C) (Figure 5).

This method allows the production of chitosan-based physical gels without the addition of cross-linking agents [42]. The gelling mechanism involves increasing the number of hydrogen bonds in the chitosan structure and decreasing electrostatic repulsions due to the presence of β-glycerophosphate. Subsequently, electrostatic interactions between ammonium and phosphate groups and chitosan–chitosan hydrophobic interactions occur [43]. The combination of β-GP/chitosan enhances the mechanical and viscoelastic properties of the gels while maintaining favorable mucoadhesive properties of the polymers [44].

A thermosensitive ophthalmic gel that associated dosing accuracy and ease of administration of eye drops with increased ocular bioavailability of a hydrogel was formulated by Fabiano et al. by cross-linking a solution of chitosan hydrochloride with β-glycerophosphate. The active substance added as nanoparticles was 5-fluorouracil (5-FU). In order to modulate 5-FU release, polymer mixtures were used. Chitosan was partially replaced with quaternary ammonium derivatives or thiolated derivatives. The sol–gel transition ranged from 30 °C to 35 °C. Studies showed a steady-state concentration of the active substance up to seven hours after instillation, thus increasing the ocular bioavailability of 5-FU [45].

Due to the hydrogen bonds formed with the surrounding water molecules, polyols such as glycerol or glucose create a protective layer of hydration around the polymer chains. This layer remains stable at neutral pH and low temperature. It reduces interactions between polymer chains and prevents the formation of a macromolecular gel. The temperature increase causes the transfer of the proton from chitosan to the gelling agent. Thus, the degree of ionization of chitosan lowered and fewer electrostatic interactions occur between the polymer and the gelling agent. When the temperature rises, the degree of agitation of water molecules increases too but the number of hydrogen bonds decreases. Hydrogen bonds and hydrophobic interactions which appear between the polymer chains lead to gelation. Increasing the temperature is favorable for a more stable protective layer of hydration. Its rupture requires a higher thermal energy [46]. The purpose of a study initiated by Chen et al. was to develop an in situ gel with thermosensitive and mucoadhesive properties, using chitosan and glucose-phosphate disodium (DGP), for ocular delivery of levocetirizine dihydrochloride. Addition of DGP increased the pH of the chitosan solution to a physiologically acceptable range (6.8–7.2) without producing spontaneous precipitation because of the neutralizing effect of phosphate ions. The preparation being liquid at room temperature could be easily instilled in the eye. Electrostatic interactions between phosphate residues of DGP and chitosan amino groups, as well as the separation of chitosan chains by the pyranose ring, prevented the formation of a gel at low temperature and neutral pH. By heating, the hydrogen bonds between the polymer and water became unfavorable and hydrophobic interactions took place [47].

Obtained by the hydrolysis of collagen, gelatin is a biocompatible and biodegradable protein. Its advantages include low immunogenicity, solubility in water and a sol–gel transition at 30 °C [48]. Due to its low mechanical properties, it requires the addition of cross-linking agents [49]. The polyelectrolyte complex based on chitosan and gelatin may exist at a pH value below 4.7, which is the isoelectric point of gelatin [21].

Cheng et al. formulated a thermosensitive chitosan/gelatin/glycerophosphate injectable gel as a sustained release system for latanoprost in the treatment of glaucoma. The latanoprost polymer solution turned into gel in one minute at 37 °C and remained liquid for 15 min at 25 °C [50]. The sol–gel transition for chitosan/gelatin/β-glycerophosphate mixtures was the result of electrostatic repulsions between positive charge chitosan chains and electrostatic attraction between the chitosan ammonium group and the phosphate group of glycerophosphate [51].

**Figure 6:**
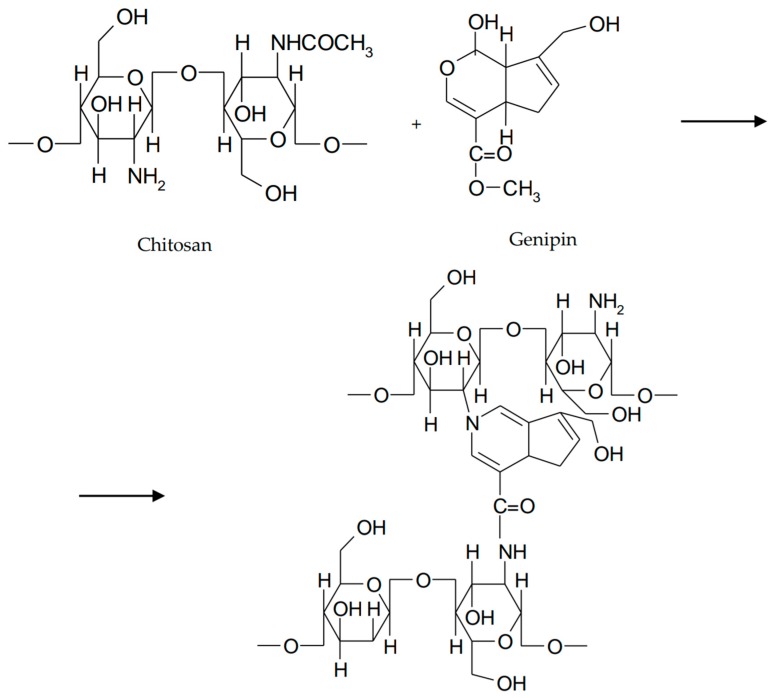
Cross-linking of chitosan with genipin.

Provided from *Gardenia jasminoides*, genipin is the hydrolysis product of geniposide [52]. Genipin has replaced glutaraldehyde and other cross-linking agents due to its stability, biocompatibility, and safety [53]. Studies have shown that implants based of chitosan and genipin exhibit good ocular tolerability on the rabbit eye [54]. In order to increase ocular retention time, Song et al. have proposed to investigate an in situ thermosensitive gel based on chitosan and gelatin co-crosslinked with β-glycerophosphate and genipin for sustained release of timolol maleate. A reduction in gelation time was observed due to the formation of a large number of hydrogen bonds between the amino and hydroxyl groups of chitosan and the hydroxyl groups of gelatin. Hydrogen bonds between gelatin and water molecules favored gel formation. To reduce the release rate of timolol, the mixture was cross-linked with genipin. Gelation time after the addition of genipin decreased significantly. Thus, the chitosan/gelatin/β-GD/genipin mixture was liquid at 4 °C and 25 °C but turned to gel at 37 °C. The cross-linking mechanism consisted of different interactions between genipin sites and the amino groups of chitosan (Figure 6) [51].

**Figure 7:**
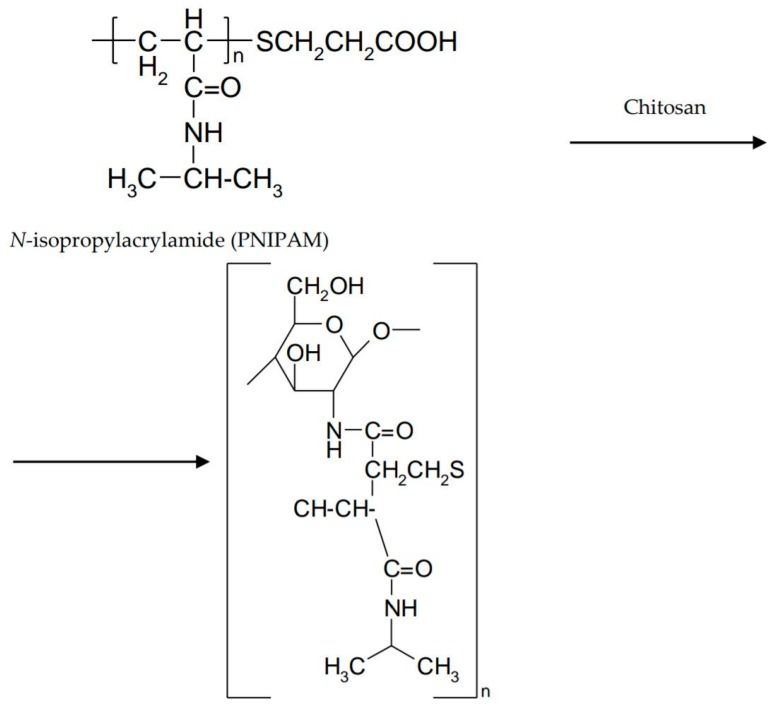
Poly (*N*-isopropylacrylamide)-chitosan interaction.

In order to improve the mechanical properties, interpenetrating polymer networks (IPN) were prepared by mixing natural and/or synthetic polymers. These biocompatible, biodegradable, non-toxic networks have gained a special place in the area of controlled drug release [55]. IPNs consist of an in situ preparation where the reactants are mixed in a solution before the cross-linking takes place. The combination of natural and synthetic polymers, as well as grafting of natural polymers on synthetic ones, determines a wide range of properties [56]. Some synthetic polymers have been associated with chitosan to form thermosensitive IPN hydrogels [12]. Poly (*N*-isopropylacrylamide) (PNIPAM) is a well-known thermo-responsive polymer. The sol–gel transition occurs around 32 °C and is accompanied by a volume transition that limits its applicability. To overcome this inconvenience, a thermostable polymer can be used as a matrix in which PNIPAM is incorporated. The introduction of PNIPAM into a chitosan gel avoids volume transition [57]. A poly (*N*-isopropylacrylamide)-chitosan polymer blend was studied by Cao et al. for the ability to form an in situ thermosensitive gel with potential ocular applicability. The polymer mixture showed LCST around 32 °C, a value close to the eye surface temperature. At temperatures below LCST, the polymer molecules were dissociated. Above LCST, hydrogen bonds between the polymer and the water broke up, increasing the number of hydrophobic interactions (Figure 7) [58].

The most abundant resource, cellulose has many hydroxyl groups that can be used to prepare hydrogels with interesting structures and properties [59]. Cellulose derivatives used in ophthalmic formulations are methyl cellulose (MC), hydroxypropyl methylcellulose (HPMC), or sodium carboxymethyl cellulose (NaCMC). At concentrations of 1–10%, they are solutions that gel after heating [60]. The aqueous solution of MC undergoes a phase transition depending on the temperature, and the addition of a salt reduces its gelation temperature [61]. MC has a phase change temperature between 40 °C and 50 °C. Sol–gel transition for HPMC is between 75 °C and 90 °C. Sodium chloride can reduce the gelation temperature at 34 °C for MC and at 40 °C for HPMC [62]. Cellulose derivatives, especially ether ones, are used as bioadhesives. The factors affecting the adhesion strength are the ability to extract water from mucus and the pH at the site of action [63]. Chitosan hydrogels have low mechanical strength, and this disadvantage limits their applicability in topical semisolid forms. In order to increase the mechanical resistance of chitosan gels, it has been suggested to add cellulose derivatives which are hydrophilic polymers [64]. Gupta et al. associated chitosan with HPMC in order to develop a sustained release system of timolol maleate in the eye. Chitosan was used as a transcorneal permeability enhancer and viscosity enhancer. It also had the ability to convert to gel at ocular pH. HPMC was a viscosity agent that reduced chitosan concentration in order to obtain transparent gels [65]. Kashikar et al. formulated and evaluated an ocular delivery system of ofloxacin based on the association of Pluronic F-127 and Pluronic F-68, along with chitosan as a permeability enhancer. Addition of HPMC led to rapid and long-lasting gelation. The addition of two mucoadhesive polymers resulted in a lowering of gelation temperature for the in situ gels. Chitosan had the capacity to form hydrogen bonds with polyoxyethylene units from poloxamer structure and dehydration took place. This phenomenon determined a higher entanglement of molecules and a lower gelation temperature [66]. Ahmed et al. proposed to incorporate polylactide-co-glycolide nanoparticles (PLGA) with ketoconazole into polymeric in situ gels for the treatment of ocular fungal infections. HPMC was added to all preparations. Chitosan and HPMC formulations had the longest gelation time. Addition of HPMC to chitosan-based formulations increased the viscosity after dilution with simulated lacrimal fluid [67].

### 2.3. Ion-Sensitive Ocular In Situ Gels Based on Chitosan

Ion-sensitive systems are known as osmotically triggered in situ gels where the polymer undergoes a sol–gel transition due to ion concentration changes, especially mono and divalent ones such as Na, Mg, or Ca, present in the tear film [60]. Rising the production of tear fluid causes a dilution of viscous solutions which generates an increase in cation concentration which, consequently, enhance the viscosity of the preparation [68].

**Figure 8:**
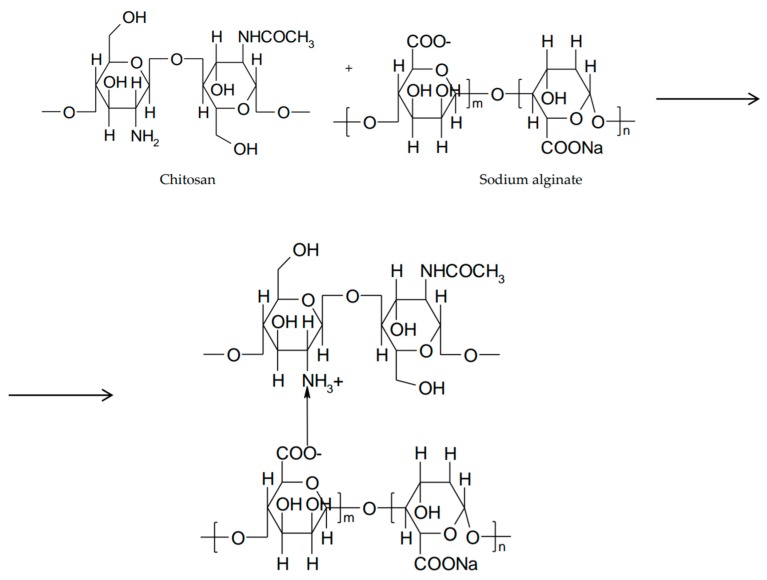
Hybrid hydrogel between chitosan and sodium alginate.

Alginic acid and sodium alginate are natural polysaccharides extracted from brown algae, made up of linear chains of β-D-manuronic acid (M) and α-L-guluronic acid (G) linked by β-1,4- and α-1,4-glycosidic bonds. In acidic medium, sodium alginate is converted to alginic acid by protonation of the carboxyl groups of manuronic and guluronic monomers. Alginic acid exists in unionized form and can generate interpenetrating polymer networks (IPNs) by hydrogen bonds. If the ratio G/M > 1, sodium alginate can form acid gels [69]. The application of alginate in ophthalmic in situ gels is preferred because of their efficiency compared to solutions. The systems are based on in situ gelling properties due to increase in their guluronic acid content, so many experiments have been conducted in vitro on simulated lacrimal fluid and in vivo on rabbit eyes. In order to enhance mechanical stability and erosion strength in different biological fluids, the surface of the alginate gel was modified by the addition of macromolecules such as chitosan or its derivatives which are capable of establishing ionic bonds with the carboxyl groups of alginate. This creates a shell around the alginate gel that becomes more resistant (Figure 8) [70].

The objective of a study initiated by Gupta et al. was to develop an in situ gelling system based on sodium alginate and chitosan for ocular delivery of levofloxacin and comparing it with conventional ophthalmic drops. Chitosan was insoluble in neutral and alkaline medium but turned into gel when pH reaches 7.4. Sodium alginate converted into a gel upon contact with divalent cations present in simulated lacrimal fluid. The dual mechanism provided a gel with good rigidity and pseudoplastic behavior. Studies showed that eye drops were rapidly removed from the corneal surface and entered into the systemic circulation, while the in situ gel showed a slow rate and prolonged retention time on the corneal surface [71].

A study by Mehra et al. intended to increase the ocular bioavailability of pilocarpine by formulating an in situ gel with sodium alginate and added tamarind gum and chitosan to increase the mucoadhesive properties. Chitosan- based systems had a sustained release of the active substance, although to a smaller extent than tamarind gum. Due to the amino and hydroxyl groups, chitosan interacted with the negatively charged mucin, enhancing the ocular bioavailability of the active substance [72]. Chitosan may form hybrid hydrogels by cross-linking with biocompatible functional polymers [12]. Xu et al. attempted to synthesize an in situ injectable gel based on glycol chitosan and oxidized alginate in order to encapsulate Avastin for ocular delivery. The hydrogel structure was the result of the interaction between the amino group of chitosan and the aldehyde group of the alginate by forming a Schiff base. Chitosan glycol instead of chitosan was used due to its better solubility. By modulating the ratio of glycol chitosan and oxidized alginate, the gelation time of the system varied between 10 s and 5 min. The concentration of oxidized alginate influenced the rate of Avastin release. According to the researchers, in situ injectable gel is a versatile delivery system for Avastin in the treatment of posterior segment disorders [73].

Gellan gum is a natural anionic polysaccharide produced by the fermentation of *Pseudomonas elodea* and consists of repetitive units of glucuronic acid, ramnose, and glucose residues in a molar ratio of 1:1:2. Gellan gum forms stable gels in the presence of mono and divalent cations present in the lacrimal film and it is used in ophthalmic in situ gels formulation [74]. It is commercially available under the name of Gelrite^®^ [75]. Gellan gum contains hydroxyl and carboxyl functional groups through which hydrogen bonds and /or electrostatic interactions with other polymers can be formed [76]. Gels can be obtained in the tear film even when the polymer concentration is very low. Sodium proves to be the best gel-forming promoter. When drops of gellan gum solution are instilled, lacrimal film dilution occurs, but an elastic "skin" that keeps the compact drops is immediately formed [70]. Gupta et al. developed an ocular delivery system of timolol maleate based on the in situ gelling concept. Chitosan, a pH-sensitive polymer that also acted as a permeation enhancer, and gellan gum, an ion-sensitive polymer and a gelling agent, were used. According to the authors, the system consisting of 0.25% chitosan/0.5% gellan gum allowed easy instillation as drops that then underwent a sol–gel transition. The pH provided gelling of chitosan, and the ions in the tear fluid allowed the gelation of gellan gum. The advantages of this formulation compared to ophthalmic drops were increased transcorneal permeability and prolonged corneal retention time [77]. In a similar manner, Imam et al. formulated an in situ gel based on a mixture of chitosan, gellan gum, and polyvinyl alcohol (PVA) for ocular release of besifloxacin. Before instillation, the system was a solution which, in contact with the simulated tear fluid, turned into a transparent gel. The phase transition of the system took place on the surface of the cornea and was the result of a triple mechanism. Chitosan acted as a mucoadhesive and penetration enhancer and had a phase transition at pH > 6.5. PVA increased mechanical strength of the system, and gellan gum was ion-dependent [78].

## 3. Evaluation of Ocular In Situ Gels Based on Chitosan

Ophthalmic preparations should be extremely pure and free of physical, chemical, or biological contaminants. They should be formulated and packaged for instillation into the eye. These requirements imply an increased responsibility in the pharmaceutical industry to maintain good manufacturing practices (GMPs) in the preparation of ophthalmic forms. According to the International Organization for Standardization, quality control represents the operational techniques and activities used to meet the quality requirements. After finishing the preparation process, control tests are carried out for the qualitative and quantitative assessment, accompanied by the procedures and limits of acceptability that the final product has to perform [79].

Ophthalmic in situ gels must be biocompatible, safe, biodegradable, and without side effects. The formulations must have pseudoplastic behavior with thixotropic characteristics. This allows easy spreading on the ocular surface by blinking and transformation into a viscous fluid by favoring a prolonged ocular retention time [68].

### 3.1. Visual Appearance and Clarity

Physical appearance and clarity are determined by visual examination before and after gelling, alternatively, against a black or white background. It is noticed whether unwanted particles or possible opalescence are in the solution [80]. An ideal in situ gelling system should be a transparent solution at temperatures between 4–25 °C and should be transformed into a clear gel at temperatures between 30–37 °C [81]. The formulations proposed by Kashikar et al. based on chitosan, poloxamer, and HPMC for ocular delivery of the ofloxacin, exhibited transparent gels after instillation into the eye that spread easily on the eye surface [66]. A formulation consisting of 0.5% chitosan and 0.2% sodium alginate was a transparent and colorless solution at pH 6 that turned into gel at ocular pH, providing prolonged levofloxacin retention time without interfering with the vision process [71]. At high concentrations, chitosan does not generate a clear solution, so that after instillation, a white precipitate is formed which disturbs vision due to precipitation of chitosan at pH 7. To avoid this inconvenience, Gupta et al. have reduced the concentration of chitosan by the addition of HPMC, a viscous agent, and obtained a suitable in situ gelling system for ocular delivery of timolol maleate [65]. The addition of PVP to a polymer solution of chitosan-poloxamer changed the color to bright yellow [82]. The mechanism of genipin cross-linking of an in situ gel based on chitosan and gelatin produced a series of reactions between different sites of the genipin structure and the primary amino groups of chitosan and gelatin, causing a blue coloration of the gel. Depending on the concentration of genipin, the color may change from bluish to blue [51].

### 3.2. pH

It is of critical importance the effect of pH on solubility and stability. The pH of an ophthalmic formulation should ensure its stability and at the same time should not be irritating to the patient after administration [83]. The pH of the tears is approximately 7.4. The eye can tolerate formulations whose pH varies between 3.0 and 8.6, depending on the buffer capacity of the preparation. The pH value of the formulation should be that at which the active substance is the most stable. Pharmaceutical forms approaching the extreme values of the acceptability interval have greater patient tolerance if they have a low buffer capacity [84].

The pH of in situ gelling systems after the addition of all components is measured using a digital pH meter [83]. Also, the pH of solutions or in situ gels can be determined by a potentiometric method. This is the measurement of the potential difference between the electrodes placed in the examined solution and the reference solution or between the glass electrode and the reference electrode (calomel, silver), both found in the test formulation [85]. Chitosan is a pH dependent cationic polymer which remains dissolved in aqueous solution up to a pH of 6.2 [86]. When β-glycerophosphate, a weak base, is added, chitosan remains in solution at neutral pH and at room temperature. A homogeneous gelation of the system occurs on heating [42].

### 3.3. Gelation Studies

The main interest for in situ gels is the prolonged release with increased ocular bioavailability of active substances. Gelation must occur rapidly to avoid elimination of the preparation in liquid form. The phase transition of sol–gel takes place under the influence of three main stimuli depending on the polymers used [87]. An optimal in situ gel which comprises a thermosensitive polymer must have a gelation temperature higher than room temperature and shifts to gel at a temperature of 35 °C after mixing with artificial tears [88]. Ur-Rehman et al. evaluated the phase transition temperature for an in situ gel system of poloxamer, chitosan, and sodium tripolyphosphate (TPP) by the visual tube inversion method. Vials containing 1 g of sample were placed in a water bath where the temperature was gradually increased. The temperature where the sample did not flow, was noted as the gelation temperature (t_1_). Subsequently, the sample was placed in a hot bath which was gradually cooled. The temperature at which the sample started to flow was noted as gel melting temperature (t_2_). A digital thermometer was placed in a similar vial containing 1 mL of water and situated next to the sample vial in the water bath. Three measurements t_1_ and t_2_ were performed for each sample, which was prepared and analyzed in duplicate. The gelling temperature was calculated as the measured value for t_1_ and t_2_ ± SD (standard deviation) [89].

Another method for determining the gelation temperature was used by Varshosaz et al. for an in situ gelling system based on poloxamer and chitosan. A 10 mL sample and a magnetic stirrer were placed in a transparent vial which was put in a thermostatic water bath. An accurate 0.1 °C thermometer was immersed in the sample. The solution was heated at a rate of 2 °C/min under continuous stirring of 500 rpm. Gelation temperature (GT) was determined when the magnetic stirrer stopped rotating due to gelation. Each sample was measured in triplicate [41].

Furthermore, to assess the phase transition temperature after dilution, measurements were made at temperatures between 15 and 37 °C. The sol–gel transition was determined by measuring the shear stress at 500 rpm and the temperature was increased with 4 °C every 10 min. The gelation temperature was noted as the point at which a sudden change in shear stress was observed. Dilution with simulated lacrimal fluid was performed in a ratio of 40:7. It is believed that the average volume of an instilled drop in the eye is approximately 40 μL and the available lacrimal fluid volume is 7 μL [82].

The ionic concentration of the lacrimal film is a major criterion for achieving gelation in the case of ion-sensitive polymers. Thus, simulated tear fluid (STF) needs to be involved in gelation tests [87]. Several researchers have described in their studies the composition of simulated lacrimal fluid used as shown in Table 1 [37,40,82].

Imam et al. have conducted In vitro gelation studies for a chitosan and gellan gum system using simulated tear fluid. The polymer solution was mixed with STF in a ratio of 90:10. It has been observed that the gel strength is dependent on the concentration of the polymers used [78].

Balasubramaniam et al. compared the STF effect shown in Table 1 with a solution supplemented with lacrimal proteins such as albumin, lysozyme, and γ-globulin. The result was that there were no differences between the two compositions in terms of action on the gelation process, suggesting that the role of the proteins was insignificant in that process [90]. However, there are authors who use protein-based STF formulations such as that from Table 2 [91].

Performing gelation studies for in situ gels based on pH-sensitive polymers such as chitosan and Carbopol were made by placing a drop of the assay formulation in a vial containing a definite volume of STF or phosphate buffer heated to 37 °C. The researchers followed the visual inspection of the gel, gelation time, and gelling capacity according to the code in Table 3 [25,87,92].

### 3.4. Rheological Characterization

Increasing the viscosity of a formulation improves the retention time at the ocular surface. The inclusion of the rheological assessment in a product’s specifications should be based on the type of formulation and if the changes in the viscosity affect the performance of the product. It is not a compendial test but it is part of the manufacturer’s specification [84]. Zaki et al. concluded that the retention on the corneal surface of a product begins to increase when the viscosity of the fluid exceeds the critical value of 10 mPa·s. Values above 100 mPa·s prolong contact time but cause discomfort due to an increased shear stress during blinking [93]. The tear film has a viscosity of 1.5 mPa·s, but it is a non-Newtonian fluid due to the presence of mucin and other macromolecules. According to Zhu and Chauhan, any variation in viscosity below 10 mPa·s leads to imperceptible changes in the lacrimal drainage rate [94].

The rotational viscometer is the most used viscometer, but it provides relative viscosity measurements. In addition, gel behavior is not observable at different shear rates. However, it is used extensively because it allows an overview of viscosity [87]. Yu et al. determined the rheological properties of ophthalmic in situ gels based on carboxymethyl chitosan and poloxamer, cross-linked with glutaraldehyde, using a rheometer. A temperature ramp in oscillatory mode was performed using cone-and-plate geometry between 20 °C and 40 °C. When the temperature rose, the viscosity of the hydrogels increased, too. The temperature at which the viscosity had a sharp increase was noted as the gelation temperature [95].

Rajalakshmi et al. prepared in situ gels based on chitosan and poloxamer in order to enhance the ocular retention time of gemifloxacin mesylate. They evaluated the rheological profiles using the Brookfield viscometer. The formulations were placed in a sample tube. The samples were analyzed at room temperature, then heated to 37 ± 0.5 °C by a thermostatic circulating bath connected to the viscometer. The angular velocity of the spindle was increased from 10 to 100 and the viscosity was measured. The formulations showed a Newtonian flow before gelation, and after gelation, they had pseudoplastic behavior [96].

The measurement of the storage modulus (G’) and the loss modulus (G”) is preferred because it allows an accurate determination of the viscoelastic behavior and the presence of the gel status [87]. Krtalić et al. developed ophthalmic in situ gels based on chitosan, poloxamer P407 and poloxamer P188, modulated in terms of polymer concentration and rheological properties. For rheological measurements, a parallel plate rheometer was used. G’, G”, and dynamic viscosity (η*) were recorded in a temperature range from 5 °C to 85 °C. Frequency sweep measurements for the prepared mixtures indicated that G’ was greater than G”, suggesting a gel stability. Rheological profiles exhibited the existence of soft gels due to the moderate concentration of poloxamers. High values of G’ and G” were due to the presence of chitosan in the system. They revealed high mechanical strength of gels [8].

### 3.5. In Vitro Drug Release

In vitro release tests are valuable tools for monitoring the release of active substances from semisolid formulations following product development and quality control [97]. In the relevant pharmacopoeias there are no compendial apparatus, procedures or requirements for in vitro release testing [98]. Based on the FDA guidance, the method of determining In vitro release is performed using the Franz diffusion cell type system, having a synthetic polymeric membrane. The membrane separates the donor compartment containing the sample from the receptor compartment filled with medium, usually PBS buffer. Drug diffusion from the product and through membrane is monitored by analyzing samples, sequentially collected from the receptor medium. At predetermined times, an aliquot is taken from the medium, and the same amount of fresh medium is refilled to the receptor compartment to maintain a constant volume. Analysis of the active substance content is done by high pressure liquid chromatography (HPLC) or other analytical techniques [99].

A study aimed comparing a number of anionic polysaccharides—such as gellan gum, xanthan gum, carrageenan, alginate, and HPMC—with cationic polymers such as chitosan. Among the tests performed was in vitro release for ophthalmic in situ gel systems. Researchers used a standard thermostated Franz diffusion cell. The receptor chamber was filled with simulated tear fluid and stirred constantly at 600 rpm. The donor chamber, in which was placed a determined volume of the formulation containing pilocarpine, was separated from the receptor by a dialysis membrane soaked in the receptor medium. Studies showed that when gel viscosity increased, the rate of drug diffusion decreased through the gel matrix. Cations from receptor medium diffused in the matrix of anionic polymers favoring gelation. Chitosan had the highest release rate. This was the result of the lack of interactions between chitosan and the cations from the simulated tear fluid, as well as repulsions between the chitosan-positive amino group and pilocarpine hydrochloride [100].

The assessment of the in vitro release of levofloxacin microspheres from a thermosensitive chitosan gel was performed using a dialysis bag method. The samples to be analyzed were placed in dialysis bags which were soaked in phosphate buffer (pH 7.4) at 37 °C. After gelation in dialysis bags, a determined volume of phosphate buffer solution was transferred to individual tubes at predetermined time intervals. After each sampling, an equivalent volume of fresh medium was added. For each sample, levofloxacin absorbance was read at spectrophotometer. The chitosan gel favored the slow release of levofloxacin after 15 min, preventing drug burst release in the initial phase [101]. Other authors studied in vitro drug release from in situ gels based on chitosan, poloxamer, and tripolyphosphate (TPP) using both polycarbonate permeable membrane systems and membrane-free systems. For membrane-free systems, the drug release profile of gels was studied in water-rich environments such as the eye. Studies showed that chitosan and poloxamer gels had a higher dissolution rate than poloxamer gels and chitosan-poloxamer-TPP gels. The gel formed by chitosan in the presence of TPP interpenetrated the polymeric network of poloxamer. This reduced the rate of penetration of water into the poloxamer gel, delaying the unpacking of the poloxamer micelles and the subsequent dissolution of the gel. In both models, the researchers observed that the chitosan-poloxamer-TPP gels did not completely dissolve at the end of the experiments compared to poloxamer gels, and drug release from the chitosan-poloxamer-TPP gels was more sustained. Chitosan-TPP association prevented the dissolution of the poloxamer gel and favored sustained release of the drug [89].

Varshosaz et al. assessed the ciprofloxacin release from in situ gels of chitosan and poloxamer by bringing the samples into circular plastic containers and placing them in a beaker filled with STF, thermostated at 37 °C and stirred at a rate of 20 rpm. They took aliquots from the release medium at predetermined time intervals. It has been observed that an increase in the concentration and molecular weight of chitosan caused an increase in gel resistance. The mechanism involved reducing number and size of water channels and rising the number and size of micelles in the gel structure. Ciprofloxacin release from in situ gels was performed according to the Higuchi model, and data analysis showed that all gels delivered the active substance by Fickian mechanism [41].

### 3.6. Sterility Tests

Sterility is a mandatory requirement for ophthalmic formulations. Contaminated preparations produce ocular infections that can cause blindness especially if *Pseudomonas aeruginosa* is involved [79]. The European Pharmacopoeia requires that materials and methods used for the preparation of ophthalmic forms ensure sterility and avoid the introduction of contaminants or growth of microorganisms. Storage should be done in a sterile, tamper-proof container [102]. Antimicrobial agents should be added to products stored in containers that allow multiple doses only if there are no situations such as: the substance contains a radionuclide with a physical half-life of less than 24 h or the drug has antibacterial action without additives [84].

Agar diffusion test was used by Imam et al. to assess the antimicrobial efficacy of an ophthalmic in situ gel of chitosan, poly (vinylalcohol), and gellan gum containing besifloxacin compared with a besifloxacin suspension. The sterile agar petri plates were incubated with test organisms: *Pseudomonas aeruginosa* and *S. aureus*. In situ gel showed antimicrobial activity superior to the suspension after 24 h [78]. The same method was used by Bhoyar et al. for assessing the antimicrobial activity of an ophthalmic in situ gel of chitosan, poloxamer, and poly (vinylalcohol). The researchers analyzed the effect of ciprofloxacin on Staphylococcus strains, measuring the diameter of the inhibition zone for gel and eye drops. Greater inhibition zone values were obtained for gels compared to standard solutions. This was due to prolonged diffusion of ciprofloxacin from the polymer mixture [82].

An in situ gel containing 1% chitosan and 15% poloxamer with gemifloxacin mesylate did not show any turbidity and evidence of fungal growth when incubated for at least 14 days at 20–25 °C in soybean casein digest medium [96]. The antifungal activity of ocular in situ gels with ketoconazole was tested on *Candida albicans* strains using agar diffusion technique. A large diameter of the inhibition zone was observed in the formulation based on chitosan and alginate [67].

In the literature, it is cited the antibacterial and antifungal activity of chitosan, an additional benefit in the preparation of ophthalmic forms. The main mechanism of action is alteration of bacterial or fungal cell permeability due to interactions between positive charge chitosan and negative cell membrane. Antimicrobial action is superior to gram positive bacteria compared to gram negative bacteria that additionally have an external membrane [103,104,105,106].

### 3.7. Ocular Tolerability

In order to reduce the risk of exposure to harmful substances, all products manufactured for instillation into the eye, as well as their ingredients, must be tested and evaluated for the potential of ophthalmic irritation. Ocular toxicity tests are designed to ensure that the risks associated with products fulfill the safety criteria [107]. Draize rabbit eye irritation test is the oldest eye irritation test developed by Draize et al. in 1944. It continues to be widely used and approved by the Organization for Economic Cooperation and Development as well as by the FDA [108]. The procedure involves applying 0.1 mL (0.1 g solid) test substance to the cornea and cul-de-sac of a rabbit eye for up to 72 h while the other eye serves as a control [107]. The response of validation studies not only depends on the performance of the In vitro method, but also on the quality and variability of in vivo data that often serve as a reference for comparison [109].

Rajalakshmi et al. evaluated the potential ocular irritation for an in situ gel which comprised chitosan and poloxamer following the Draize test protocol. Studies were conducted on two male rabbits weighing 1.5–2 kg. The sterile formulation was instilled twice daily for seven days, and the rabbits were periodically monitored for redness, inflammation, or ocular swelling. The formulation containing 15% poloxamer/1% chitosan did not cause irritation or abnormal clinical signs in the cornea or conjunctiva [96]. In situ gels based on chitosan and gelatin co-cross-linked with disodium β-glycerophosphate and genipin were evaluated using the modified Draize test. In this case, six rabbits were used for the test and they received 50 μL of the sample in the conjunctival sac. The animals were monitored for congestion, inflammation and conjunctival redness up to 24 h after instillation. The result of the test showed lack of ocular irritation or inflammation and sustained the good biocompatibility and ocular tolerance suitable for ocular application [51]. However, it is important to note that there are remarkable physiological differences between rabbit and human models, particularly with respect to the blinking rate that is lower in rabbits. Also, the thickness of the cornea is another important factor to consider. It is thinner in rabbits than in humans [110].

An alternative to the Draize test is the chorioallantoic membrane (HET-CAM test), a sensitive and not expensive method, that uses fertilized hen eggs. The HET-CAM test has a good correlation with in vivo ocular irritation. The membrane which separates the embryo from internal airspace is non-innervated, highly vascularized, and responds to the lesion in a manner similar to rabbit conjunctiva [111]. The test provides qualitative information on potential conjunctival effects, while coagulation assessment can be used as a reflection of potential corneal lesions. The degree of irritation varies with the classification system used and can be registered according to a scoring scheme such as that from Table 4 [40,107].

Incubated egg testing is a borderline between in vitro and in vivo that does not conflict with ethical and legal standards. In situ gels of chitosan and gellan gum were tested using this method and the results were compared with those obtained using a saline solution. The formulations proved to be non-irritating to slightly irritating, and the average score was 0.67 after 24 h [77].

In vitro toxicity tests and cell culture assays have advantages over in vivo testing methods because they are inexpensive, simple, and easy to handle. They also allow an understanding of the cellular or molecular toxicity. Some tests associate cell staining with fluorescence or absorbance measurement to study changes in cell number and to determine whether a substance is cytotoxic [107].

Chitosan and gelatin are natural polymers with good biocompatibility and biodegradability [41]. Tsai et al. evaluated the effects of a thermosensitive gel of chitosan, gelatin, β-glycerophosphate with ferulic acid for corneal wound healing using fluorescein staining. A rabbit corneal alkali burn model was used. The results proved that the gel significantly reduced the surface of the injured area after the first three hours. After 24 h, the histological analysis showed a slight corneal hyperplasia [112]. The deacetylation degree of chitosan is closely related to its biocompatibility. Thus, when the degree of deacetylation of chitosan is higher, the ocular retention time is prolonged and no inflammation is detected. Cheng et al. developed a thermosensitive gel for ophthalmic application based on gelatin and chitosan with a deacetylation degree over 95%. The hydrogel cytotoxicity was assessed on human corneal epithelial cells using crystal violet staining method. The results showed that there were no harmful effects of the ophthalmic gel containing latanoprost on the cells [50].

Enzymatic assays are common methods for evaluating ocular cytotoxicity. These include MTT assays (3-(4,5-dimethylthiazol-2-yl)-2,5-diphenyltetrazolium bromide, or MTT) that measure the reduction of yellow MTT to purple formazan under the action of mitochondrial succinate dehydrogenase. The change of color can be measured spectrophotometrically and allow the estimation of cell viability [107]. To evaluate the biocompatibility and viability of cells following instillation of an ophthalmic in situ gel based on hexanoyl glycol chitosan, Cho et al. used the live/ dead assay in addition to MTT test on both corneal epithelial cells and human conjunctival cells. The researchers found that the proliferation rate of gel-treated cells was not significantly different from that of cells that did not come in contact with the gel. However, the viability of cells exposed to the gel was assessed by live/dead assay. Most cells treated with the hexanoyl glycol chitosan gel survived for two days. This suggested that the proposed ophthalmic gel was biocompatible and that it could be a suitable candidate for ocular release of brimonidine tartrate in the treatment of glaucoma [113].

## 4. Conclusions

Ophthalmic approaches are always a challenge because they have to overcome the problems associated with conventional eye drops. In situ gels are a promising alternative as they increase the retention time and the ocular bioavailability of the active substances. In situ gels also combine advantages of solutions such as accuracy and reproducibility of dosing, or ease of administration with prolonged contact time, a characteristic of ointments. At the same time, decreasing the frequency of administration increases the patient’s compliance. A natural polymer, chitosan is often included in ophthalmic formulations due to its biocompatibility and biodegradability, permeation enhancing effect, corneal wound healing effect, and antimicrobial and antifungal actions. The prolonged contact time on the ocular surface is due to its mucoadhesive nature. The positively charged amino groups of the chitosan structure interact with the negatively charged mucinous layer. Chitosan in situ gels show high sensitivity to pH changes. The association of chitosan with stimuli-responsive polymers increases the mechanical strength of the formulation, resulting in better compliance and increased therapeutic effect.

The eye is an extremely sensitive organ, so the safety of ocular formulations is a very important criterion. Identifying the optimal concentration ratio between the associated polymers influences the appearance, pH, viscosity, and in vitro drug release. Thus, a critical control of the viscosity value is required because a high value may interfere with the vision process, producing blurred vision or discomfort. Ocular tolerability studies indicate the absence of abnormal corneal or conjunctival clinical signs after application of chitosan-based in situ gels.

Currently, most chitosan in situ gels are intended to deliver a single active substance, so that in the future it would be desirable to create new strategies to associate more active ingredients or to combine chitosan with a wider range of polymers that have synergistic action in the treatment of ocular disorders.

## Figures and Tables

**Table 1:** Composition of simulated tear fluid (STF).

Ingredients	Amount (g/mL)
Sodium chloride	0.670
Sodium bicarbonate	0.200
Calcium chloride 2H_2_O	0.008
Bidistilled water q.s. ad	100

**Table 2:** Composition of protein-based STF.

Ingredients	Amount (mg/mL)
Lysozyme	2.68
D-glucose	6.50
Gamma globulin	1.34
Sodium chloride	6.50
Bovine serum albumin	2.68
Calcium chloride 2H_2_O	0.08

**Table 3:** Gelling capacity code.

Observation	Coding
No gelation	-
Gelation occured in few minutes and remained for few hour	+
Gelation immediate, remained for few hour	++
Gelation immediate, and for extended period	+++
Very stiff gel	++++

**Table 4:** Scoring chart for HET-CAM test.

Effect	Scores	Inference
No visible hemorrhage	0	Non-irritant
Only visible membrane discoloration	1	Mild irritant
Structures covered partially due to membrane discoloration or hemorrhage	2	Moderately irritant
Structures covered totally due to membrane discoloration or hemorrhage	3	Severe irritant
